# Severity of the COVID‐19 pandemic in India

**DOI:** 10.1111/rode.12779

**Published:** 2021-05-18

**Authors:** Katsushi S. Imai, Nidhi Kaicker, Raghav Gaiha

**Affiliations:** ^1^ Department of Economics School of Social Sciences University of Manchester Manchester UK; ^2^ School of Business, Public Policy and Social Entrepreneurship Ambedkar University Delhi India; ^3^ Population Studies Center University of Pennsylvania Philadelphia Pennsylvania USA; ^4^ Global Development Institute The University of Manchester Manchester UK

**Keywords:** COVID‐19, cumulative severity ratio, daily severity ratio, India, Maharashtra, random‐effects model

## Abstract

The main objective of this study is to identify the socioeconomic, meteorological, and geographical factors associated with the severity of COVID‐19 pandemic in India. The severity is measured by the cumulative severity ratio (CSR)—the ratio of the cumulative COVID‐related deaths to the deaths in a pre‐pandemic year—its first difference and COVID infection cases. We have found significant interstate heterogeneity in the pandemic development and have contrasted the trends of the COVID‐19 severities between Maharashtra, which had the largest number of COVID deaths and cases, and the other states. Drawing upon random‐effects models and Tobit models for the weekly and monthly panel data sets of 32 states/union territories, we have found that the factors associated with the COVID severity include income, gender, multi‐morbidity, urbanization, lockdown and unlock phases, weather including temperature and rainfall, and the retail price of wheat. Brief observations from a policy perspective are made toward the end.

## 1 INTRODUCTION

More than 15 months have passed since December 1, 2019, when the first case of COVID‐19 was confirmed in China (Wu et al., 2020) and more than 13 months have passed since the first positive COVID‐19 case was registered in India on January 30, 2020, in Kerala. As of March 14, 2021, the total coronavirus infection cases in India were 11,358,961 with the death number 158,644 (both of which ranked the third next to the USA and Brazil).^1^ However, there exists significant heterogeneity across different states. For instance, the state of Maharashtra alone has recorded one‐fifth of the total cases and one‐third of the total deaths.^2^ Despite rapid progress in medical research on COVID‐19, what non‐medical factors, in particular socioeconomic factors, are associated with the COVID‐19 pandemic in India remains largely unknown. Our focus in the present study is thus on the socioeconomic, meteorological, and geographical factors associated with the severity of the COVID‐19 pandemic in India. Although there has been a surge in the studies about the socioeconomic impacts of COVID‐19,^3^ few studies, to the best of our knowledge, have examined correlates of COVID‐19 infections in developing countries, including India. Our study is a modest attempt to fill the gap in the literature.

More specifically, we carry out regression analyses for the weekly and monthly panel data sets of 32 Indian states/union territories in March–October 2020 to understand the pandemic in these states in the national context to identify the correlates of the COVID‐19 pandemic.^4^ We also focus on the state of Maharashtra where the pandemic has been severest. Maharashtra, home to around 10% of the total population of India^5^ and classified as one of the richest states—based on per capita income—has recorded the highest number of cases and deaths linked to the COVID‐19 virus so far.

The research questions we propose to ask are: (1) what are the factors associated with the severity and infection cases of COVID‐19 pandemic in India? and (2) how has the pandemic of COVID‐19 developed in Maharashtra in comparison with other Indian states? Given the nature of the data (i.e., the state‐level panel data), it would be difficult to identify the causal relationship.^6^ However, even if we cannot identify the causality, it is our view that detailed analyses of the factors correlated with the pandemic development would be still useful for understanding the nature of the COVID‐19 pandemic.

The rest of the paper is organized as follows. Section 2 reviews the emerging literature on the COVID‐19 pandemic with a particular focus on the correlates of the COVID‐related deaths and illness as well as infections. Section 3 defines the severity ratios we use in the present study to capture the severity of the pandemic and offers a statistical description of the data. Section 4 specifies econometric models we use to assess the severity of the pandemic. Section 5 presents econometric results. Section 6 concludes with discussions of the main results and relevant policy issues to indicate directions for future research.

## 2 LITERATURE REVIEW

Despite a surge in the studies on COVID‐19 in economics or social sciences, they are mostly about the impact or the consequences of COVID‐19 pandemic. The empirical literature on the socioeconomic factors associated with the COVID‐19 pandemic or infections is still scarce in India or in other developing countries. This section provides a selective review of the emerging literature of the correlates of the COVID‐19 pandemic in India.

In an earlier contribution, Joe et al. (2020) conducted a detailed statistical study of factors associated with the COVID‐19 pandemic mortality in India using crowd‐sourced data to provide estimates for age‐sex specific COVID‐19 case fatality rate (CFR) and the percentage of confirmed deaths in total confirmed cases. The authors concluded that (1) males have higher overall burden, but females have a higher relative‐risk of COVID‐19 mortality in India, and (2) elderly males and females both display high mortality risk and require special care when infected. As the period that this study covers ends on May 20, 2020, well before the huge surge in COVID‐19 cases—inevitably constrained by the availability of data—there is a need for covering a more recent period.

As reviewed by Das et al. (2021), recent studies on the correlates of COVID‐19 predominantly focus on the meteorological variables (e.g., Ma et al., 2020) and few studies focus on socioeconomic correlates. After controlling for temperature and moisture indices, Das et al. have found that the living environment deprivation (in terms of housing conditions, asset possession and water access/population and household density) was an important correlate of spatial clustering of COVID‐19 hotspots in Kolkata megacity, the capital of West Bengal. While we cannot include such detailed data for our study at the national level, we control for weekly temperature and rainfall as well as the ratio of urbanization at state levels.

It is evident that socioeconomic factors influence the COVID‐19 pandemic and infections, but virtually no studies have considered them in India,^7^ particularly at the national level. An important exception is Olsen et al. (2020) who have estimated a hierarchical and multilevel model to estimate the correlates of the risk of death because of COVID‐19 in 11 states of India, considering the factors at both individual and district levels. The authors combined the National Family Health Survey for 2015/16, Census data for 2011, and estimates of COVID‐19 deaths cumulatively up to June 2020 from How India Lives. Olsen et al. found that people living in urban areas, belonging to the scheduled caste, being smokers, who are males with more exposure to activities outside home, and above 65 years have a higher risk of the COVID‐19‐oriented death. While our study cannot incorporate all the factors, it will cover a few important variables, such as urbanization, the morbidity above 60 years, and income per capita.

Acharya and Porwal (2020) have constructed the aggregate vulnerability index at state and district levels based on National Family Health Survey Data in 2015/16 with a focus on the five dimensions, namely socioeconomic condition, demographic composition, housing and hygiene condition, availability of health‐care facilities, and COVID‐19‐related epidemiological factors. Among the 10 most vulnerable states, socioeconomic condition, housing and hygiene condition, and availability of health‐care facilities contributed to the overall vulnerability index. The authors found that among the eight states that have contributed to over 80% of the confirmed COVID‐19 cases in India, as of June 17, 2020, five states had a high vulnerability index value and the remaining three had medium vulnerability (e.g., Maharashtra with 33% of the total COVID cases and the vulnerability index 0.829, the seventh from the bottom). Although Acharya and Porwal have not estimated the vulnerability index using the actual COVID‐19 data, their analysis implied the importance of socioeconomic factors, which is consistent with Olsen et al. (2020).

Our study builds on the existing literature on the correlates of the COVID‐19 in India in some important ways. First, our study extends the analysis to October 31, 2020, and thus captures the surge in the COVID pandemic. We use a measure of COVID‐19 severity, namely the cumulative severity ratio (CSR). CSR takes COVID‐related deaths over a period since the occurrence of the first death relative to deaths in a pre‐pandemic year over the same duration. The first difference of CSR is taken to capture a flow measure of the pandemic based on the new COVID‐related deaths in comparison with the deaths in a pre‐pandemic year. It helps monitor the progression of the pandemic—whether it is intensifying, weakening, or unchanged. We use panel models that allow the use of time‐invariant fixed effects.

## 3 DATA AND VARIABLES

### 3.1 Definitions of severity ratios

A new indicator “relative severity” proposed by the World Bank illustrates the unequal distribution and progression of COVID‐19 deaths across states (Schellenkens & Sourrowuille, 2020). The relative severity ratio is defined as the ratio of the total deaths attributable to COVID‐19 over a given period to the expected total deaths from all causes under the counterfactual assumption that the pandemic had not taken place over a base period of the same length. A comparison with pre‐pandemic mortality patterns provides a state‐specific measure of the severity of the pandemic. In addition to this ratio (which will be denoted as CSR), Schellenkens and Sourrowuille have defined a daily severity ratio (DSR) that tracks the progression of the severity of the pandemic in each region. To calculate the DSR, the number of COVID‐19 deaths on a particular day is divided by the expected daily deaths under the assumption of no‐pandemic, that is, annual deaths divided by 365 (in a pre‐COVID year). We have modified CSR and DSR to capture excess mortalities. CSR has been redefined as the ratio of the sum of “accumulated COVID‐19 oriented death numbers and the expected death numbers” to “the expected deaths from all causes” in a certain period. Likewise, DSR is modified as the ratio of “the sum of daily COVID‐19 death numbers and the expected daily death numbers” to “the expected daily death numbers.”

Algebraically,

$$Cumulativeseverityratio_{t}=\frac{CumulativeCOVIDdeaths_{t}+\left(\frac{No.ofdeathsinapre‐pandemicyear}{365}\times Lengthofpandemic_{t}\right)}{\left(\frac{No.ofdeathsinapre‐pandemicyear}{365}\times Lengthofpandemic_{t}\right)},$$

where

$$Lengthofpandemic_{t}=No.ofdaysbetweendateoffirstCOVIDlinkeddeathandtintheregion.$$

$$Dailyseverityratio_{t}=\frac{New\left(\mathrm{daily}\right)COVIDdeaths_{t}+\left(\frac{No.ofdeathsinapre‐pandemicyear}{365}\right)}{\left(\frac{No.ofdeathsinapre‐pandemicyear}{365}\right)}$$

The COVID‐19 data are collated from Ministry of Health and Family Welfare, India. The data on past mortality patterns are based on the state‐wise number of registered deaths in 2017 from the Ministry of Health and Family Welfare, Government of India. For the purpose of deriving CSR, the number of reported deaths in 2017 is scaled down from annual estimates to the length of the pandemic in each state, calculated as the number of days since the first death in the state till the data point (*t*), with the cutoff date October 31, 2020. For the DSR, the denominator used in the ratio is the total number of deaths in each region in 2017/365.^8^ As we discuss later, we will use as a dependent variable the first difference of CSR for the weekly panel as its level is nonstationary and both the level and the first difference of CSR for the monthly panel. Descriptive statistics of the variables are presented in Table A1.

### 3.2 Trends of severity ratios

**FIGURE 1:**
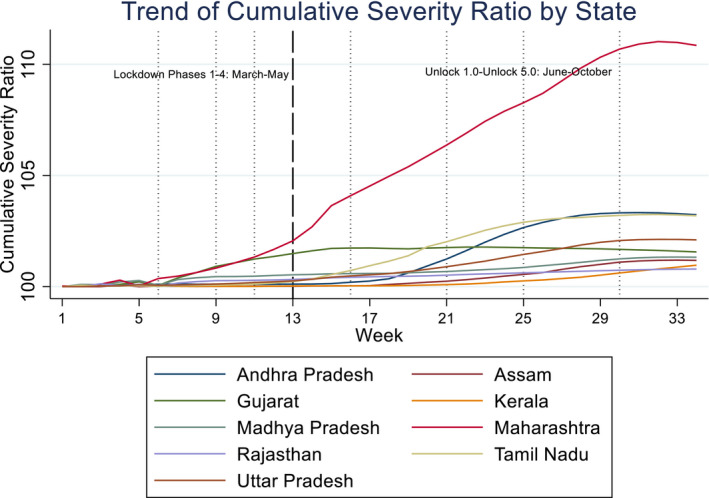
Trend of cumulative severity ratio—selected states (13–03–2020 to 31–10–2020) (%) [Colour figure can be viewed at wileyonlinelibrary.com]

**FIGURE 2:**
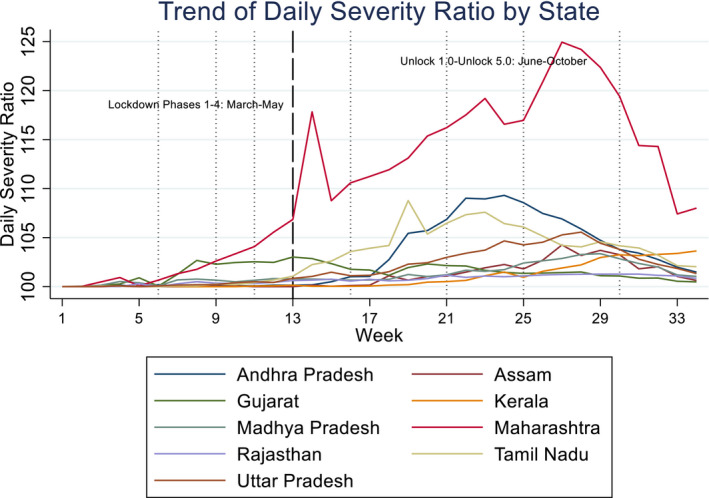
Trend of daily severity ratio—selected states (13–03–2020 to 31–10–2020) (%) [Colour figure can be viewed at wileyonlinelibrary.com]

Figures 1 and 2 show the trends of CSR and DSR, respectively, for relatively large states to avoid the graphs being cluttered. CSR and DSR are aggregated for each week, from Week 1 (starting on March 13, 2020) to Week 34 (on October 29, 2020). It is noted that during this study period the Indian government made every effort to prevent the spread of COVID‐19 starting from a rigorous lockdown policy to relatively loose restrictions. The entire period is roughly divided into “Lockdown” phases from March to May and “Unlock” phases from June to October, as indicated by a dashed line in the figures. The former is divided into four phases: Phases 1–4, and the latter into five phases: Unlock 1.0 to Unlock 5.0 as shown by dotted lines in the figures. The first lockdown (Phase 1) spanned a period of 21 days from March 25 to April 14 in which nearly all factories and services were suspended, barring “essential services.” The second lockdown (Phase 2) started on April 15 and continued until May 3, with conditional relaxations for regions where the COVID‐19 spread had been contained. With additional relaxations, the third phase of the lockdown (Phase 3) was from May 4 to May 17, and the fourth phase (Phase 4) was from May 18 to June 21.

Unlock 1.0 (June 1–30) was the first phase of the reopening in stages, with an economic focus where shopping malls, hotels, and restaurants reopened. In Unlock 2.0 (July 1–31), the lockdown measures were restricted only to the contaminated zones, and some inter‐ and intrastate travels were permitted. Further relaxation of restrictions (e.g., night curfews) occurred in Unlock 3.0, while Maharashtra and Tamil Nadu imposed a lockdown (August 1–31). Unlock 4.0 (September 1–30) was characterized by permissions of gathering at marriages/funerals, while wearing face masks became compulsory in public places and Unlock 5.0 (October 1–31) by opening cinemas and a gradual restarting of onsite teaching at schools at the discretion of state governments. How these government policies effectively influenced the COVID‐19 infection cases or fatalities is debatable and essentially an empirical question. Some authors have constructed the panel data across different countries and have estimated the effects of government policies on the COVID‐19 infections. For instance, Chen et al. (2020) estimated the effects of various non‐pharmaceutical interventions by governments to prevent the spread of COVID‐19 on the country‐level effective reproductive rate (*Rt*) for the panel of 75 economies and have found that while lockdown measures lead to reductions in *Rt*, gathering bans are more effective than workplace and school closures. How these policies are effective in India remains uncertain, which would justify our focus on different phases.

We observe in Figures 1 and 2 a gradual increase in both CSR and DSR from the latter half of Phase 1 in Maharashtra and Gujarat. However, Maharashtra has seen a continuous rise in both CSR and DSR until Unlock 4.0–5.0 where CSR exceeded 110%. DSR reached 125% in Unlock 4.0. Evidently, Maharashtra has experienced the severest pandemic. However, the state has seen a gradual decline in DSR from mid‐September to October 2020. On the contrary, CSR remained stable at around 102% in Gujarat from June to October. DSR has also remained stable in Gujarat after late July. Tamil Nadu experienced a sharp rise in CSR in July and August (Unlock 2.0–3.0). Its DSR became the second worst next to Maharashtra from mid‐June to the end of July with a gradual decline after mid‐August.

Andhra Pradesh saw a rise in CSR from early July. Its CSR became the second highest roughly at around 103% next to Maharashtra on September 18. DSR in Andhra Pradesh was the second highest in late July to early October with its peak nearly 110% in late August. DSR has declined since then. Uttar Pradesh has seen a rise in CSR from July to October. Other states in the graphs, namely Madhya Pradesh, Rajasthan, Assam, and Kerala, have experienced a gradual increase in CSR, but the pandemics measured by CSR or DSR were not as severe as those of the states mentioned earlier. We observe a large variation in levels of the severity across different states.

**FIGURE 3:**
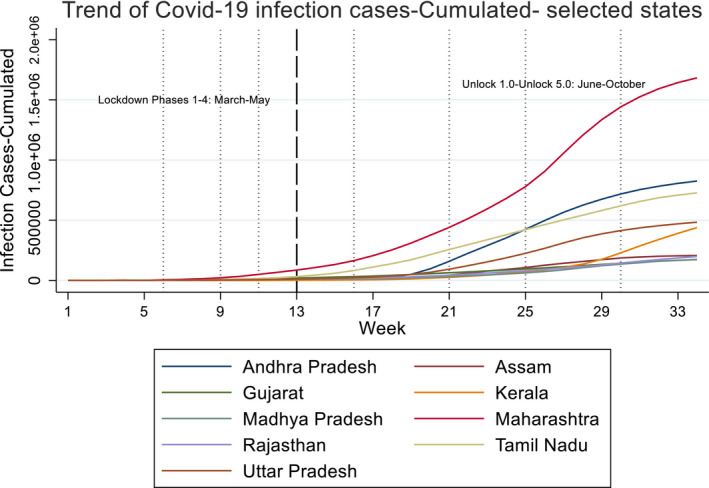
Trend of cumulative COVID‐19 infection cases (13–03–2020 to 31–10–2020) [Colour figure can be viewed at wileyonlinelibrary.com]

We see large variations in other states/union territories not highlighted in Figures 1, 2, 3. For instance, in Goa CSR increased from 102% in July to 113% in October 2020. Sikkim's CSR remained at 100% (i.e., no extra mortality due to COVID‐19) until the end of July, but its CSR suddenly rose later and reached 115% at the end of October. Puducherry showed a similar trend with a rise in CSR in August and September and CSR reached 110%. In Uttarakhand CSR gradually increased from 101% in July to 106% in October. Jammu and Kashmir saw a similar rise in CSR from 101% to 106% in July–October due to a surge in DSR in the same period. On the contrary, CSR and DSR remained very low elsewhere, such as Odisha and Mizoram.^9^

**FIGURE 4:**
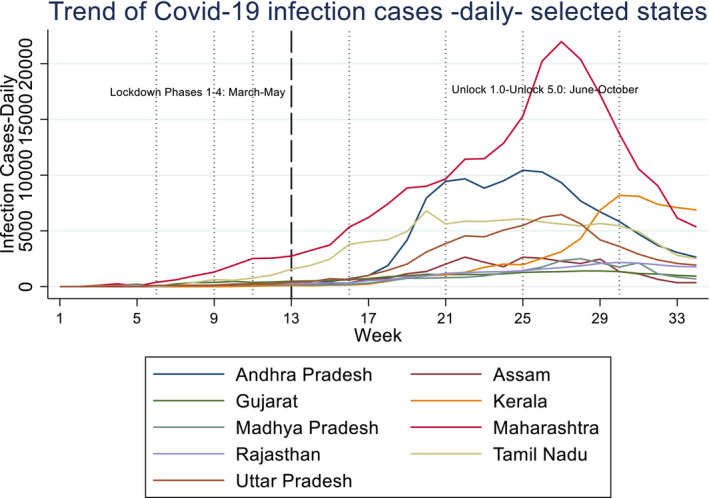
Trend of daily COVID‐19 infection cases—selected states (13–03–2020 to 31–10–2020 [Colour figure can be viewed at wileyonlinelibrary.com]

**FIGURE A1:**
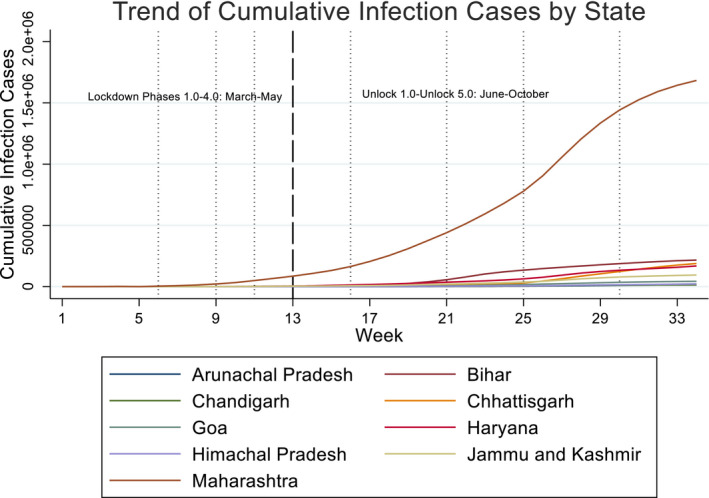
Trend of cumulative COVID‐19 infection I (13–03–2020 to 31–10–2020) [Colour figure can be viewed at wileyonlinelibrary.com]

**FIGURE A2:**
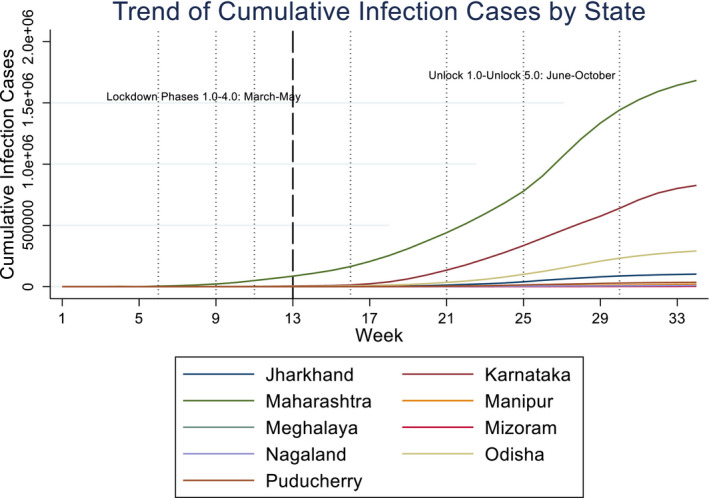
Trend of cumulative COVID‐19 infection II (13–03–2020 to 31–10–2020) [Colour figure can be viewed at wileyonlinelibrary.com]

**FIGURE A3:**
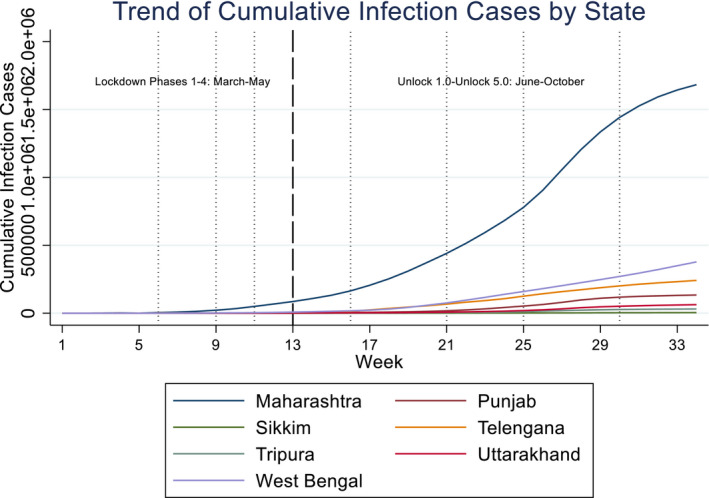
Trend of cumulative COVID‐19 infection III (13–03–2020 to 31–10–2020) [Colour figure can be viewed at wileyonlinelibrary.com]

Figures 3 and 4 show the trends of cumulative and daily infection cases of COVID‐19 on the basis of weekly averages in selected states. In terms of infection cases, Maharashtra has experienced by far the severest pandemic, although the number of daily infection cases has started to decline after September 18. On the contrary, Kerala's daily infections suddenly rose from mid‐September to the end of October—leading to a steep increase in cumulative cases. In other states shown in Figures 3 and 4, daily cases were highest in September and started to decline marginally in October. Most of the other states have shown similar trends of cumulative and daily cases where the latter declined gradually (Figures A1, A2, A3). One notable exception is West Bengal where daily cases continued to rise in September and October. The daily cases exceeded 4,000 and DSR rose to 104% in October in West Bengal.^10^

### 3.3 Panel unit‐root tests

Given the trends of severity ratios we observe in Figures 1 and 2, we have carried out unit‐root tests for CSR based on the weekly panel data. To normalize the infection cases, we have taken the logarithm of the number of cases and applied the unit‐root tests for it. Table 2 presents the results of the panel‐unit root tests for CSR, its first difference, the log of cases, as well as the log of retail prices series of wheat, one of the explanatory variables. We apply Levin–Lin–Chu (LLC) (Levin et al., 2002) and Im–Pesaran–Shin (IPS) tests (Im et al., 2003). LLC tests the null hypothesis that each time series contains a unit root against the alternative hypothesis that each time series is stationary in which the lag order is permitted to vary across individuals. IPS test is not as restrictive as the LLC test, as it allows for heterogeneous coefficients. The null hypothesis is that all individuals follow a unit‐root process against the alternative hypothesis allowing some (but not all) of the individuals to have unit roots. We apply the specifications with and without a time trend. We determine the number of lags by Akaike Information Criteria (AIC).^11^ Three states with missing observations have been dropped to make the panel balanced.

Table 1 shows that CSR is I(1) (nonstationary) but its first difference is stationary. The log of cases and the log of wheat prices are stationary. Given that CSR is not stationary, the ordinary least squares (OLS) or the static panel data model, such as fixed‐effects or random‐effects models, cannot be applied. As all the explanatory variables—including the wheat prices and weather variables—are stationary, they are not co‐integrated. Therefore, we will use the first difference of CSR or the log of infection cases as a dependent variable for the weekly panel. We have also taken the monthly averages of the data and constructed the monthly panel—where the stationarity is not an issue due to a smaller *T* (or the panel unit‐root tests cannot be conducted). For the monthly panel we use the level of CSR, its first difference, or the log of cases as a dependent variable.

**TABLE 1:** Results of unit‐root tests for weekly panel

	Levin–Lin–Chu	Levin–Lin–Chu	Im–Pesaran–Shin	Im–Pesaran–Shin
	(LLC)	(LLC)	(IPS)	(IPS)
	No trend	With trend	No trend	No trend
Panel structure				
*N* (no. of centers)	29	29	29	29
*T* (no. of periods)	34	34	34	34
Panel means	No	No	No	No
CSR				
Average lags^a^	0.9	0.86	0.93	1.62
(level)				
adjusted *t* or W‐*t*‐bar^b^	4.48	−1.43*	7.02	1.96
	I(1)	I(1)	I(1)	I(1)
CSR				
Average lags^a^	0.41	0.24	0.45	0.48
(first difference)				
Adjusted *t* or W‐*t*‐bar^b^	−15.45***	−16.57***	−14.63***	−13.82
	I(0)	I(0)	I(0)	I(0)
Log cases				
Average lags	3.72	3.93	0.68	0.79
*t* (adjusted)	−5.09***	−4.64***	−8.69***	−9.003***
	I(0)	I(0)	I(0)	I(0)
Wheat price				
Average lags^a^	0.48	0.45	0.69	0.55
(retail, log)				
adjusted *t* or W‐*t*‐bar^b^	−12.66***	−13.47***	−12.72***	−12.81***
	I(0)	I(0)	I(0)	I(0)

^a^ Lags are determined by Akaike Information Criteria (AIC)

^b^ Adjusted *t* is reported for LLC and W‐*t*‐bar is reported for IPS

* Indicates that the estimate is statistically significant at 10% level

*** Denotes the statistical significance at 1% level.

## 4 REGRESSION ANALYSES ON THE CORRELATES OF THE SEVERITY OF COVID‐19 PANDEMIC

### 4.1 Model specification

To find the explanation for the regional variation in the severity of COVID‐19 pandemic, we use a panel of 32 states/union territories for which the data on various variables are available covering the period from March 13, 2020, to October 31, 2020. As we have noted, we have organized the data as weekly or monthly panels where all the variables on daily basis are averaged for each week or month. Because of the missing observations of a few variables, our estimation is based on 1,041 observations for the weekly panel (32 states times 32.5 weeks on average) and 223 observations for the monthly panel (32 states times 7.0 weeks).

Our econometric models are motivated by the emerging empirical literature on the correlates of the COVID‐19 pandemic we reviewed in Section 2, where it has been found that not only meteorological factors but also socioeconomic and demographic factors are closely associated with the degree of the COVID‐19 pandemic or infections. We thus consider socioeconomic variables (e.g., income, multi‐morbidity, sex ratio, and urbanization) as well as meteorological and geographical factors (e.g., temperature, rainfall, and state dummy variables). Methodologically, we use pooled OLS (with both state fixed effects and phase or month dummies), a random‐effects model, and a random‐effects Tobit model so that we can model time‐invariant unobservable state or union territory characteristics (e.g., institutional or cultural factors specific to each state or union territory). In the random‐effects model, state and phase/month fixed effects are included by applying the mixed‐effects model (Bell & Jones, 2015). In the meantime, as some states/union territories had zero cases or deaths in early periods, the random‐effects Tobit model is also estimated as a robustness check to consider left‐censoring of the dependent variable in case we estimate either CSR or the log of cases.

More specifically, we regress a dependent variable, either the CSR, the first difference of CSR (DCSR), or confirmed COVID‐19 infection cases on a number of explanatory variables. CSR captures the overall development of the COVID‐19 pandemic, while its first difference denote how the severity progresses over time. We also use the number of the infection cases given that a surge in the confirmed cases is closely associated with the COVID‐19 pandemic with some lags.

We have selected the explanatory variables, while constrained by the data availability, to reflect the growing empirical literature on the correlates of COVID‐19 pandemics or infections. The time‐variant explanatory variables are weather variables, namely temperature and rainfall as well as the lagged commodity price (retail wheat price). We have also used a number of time‐invariant variables such as the log of per capita income, urbanization, presence of more than one morbidity condition among those above 60 years, and the sex ratio (the number of females per 1,000 males). The model also includes a few phase dummies as defined in the previous section.

We estimate the following equation. We have taken the logarithm of most of the explanatory variables to capture the relative effect, or the elasticity of each factor.^12^

(1) $$DCSR_{\mathrm{it}}=\beta_{0}+\beta_{1}logPerCapitaIncome_{i}+\beta_{2}Multimorbidityabove60_{i}+\beta_{3}Urbanisation_{i}+\beta_{4}SexRatio_{i}+\beta_{5}WheatPrice_{it‐1}+\beta_{6}Temperature_{\mathrm{it}}+\beta_{7}Rainfall_{\mathrm{it}}+Phase\left(\mathrm{orMonth}\right)Dummies_{t}\beta_{8}+\mu_{i}+e_{\mathrm{it}}.$$

In Equation 1 i stands for states (from 1 to 32) and t for weeks from March 13 to October 31, 2020 (from 1 to 34) for the weekly panel data and March to October (from 1 to 8) for the monthly panel data. A dependent variable is $DCSR_{\mathrm{it}}$ (the first difference of CSR) or $logCovidCases_{\mathrm{it}}$ (the log of the daily infection cases—averaged over a week)—both of which are I(0)—for the weekly panel data and $CSR_{\mathrm{it}}$, $DCSR_{\mathrm{it}},$ or $logCovidCases_{\mathrm{it}}$ (the log of infection cases averaged over a month) for the monthly panel as in Equation 2.^13^

(2) $$logCovidCases_{\mathrm{it}}=\beta_{0}+\beta_{1}logPerCapitaIncome_{i}+\beta_{2}Multimorbidityabove60_{i}+\beta_{3}Urbanization_{i}+\beta_{4}SexRatio_{i}+\beta_{5}WheatPrice_{it‐1}+\beta_{6}Temperature_{\mathrm{it}}+\beta_{7}Rainfall_{\mathrm{it}}+Phase\left(\mathrm{orMonth}\right)Dummies_{t}\beta_{8}+\mu_{i}+e_{\mathrm{it}}.$$

$PerCapitaIncome_{i}$ (PCI) denotes income at state levels that is measured by per capita net state domestic product (in Rs., divided by 1,000).^14^ PCI captures not only overall economic development at state levels. It may also capture health infrastructure or funding at state levels—for which the data are unavailable—in response to the COVID‐19 pandemic. We include the proportion of elderly people who suffer from more than one non‐communicable disease (NCDs) at state levels ($Multimorbidityabove60_{i}$).^15^ This is the proportion of population in the age group 60+ reporting more than one NCD (e.g., cardiovascular diseases, diabetes, hypertension, among others). To capture the degree of urbanization, we also insert $Urbanization_{i}$, the share of the population living in urban areas. The idea is that a higher population density and urbanization would increase more interactions among people and raise both CSR and its first difference. Furthermore, we have inserted $SexRatio_{i}$ (the number of females per thousand male), as it is well documented that, while COVID‐19 infection rates are broadly similar between men and women, men are more likely to suffer from severe illness or die as a result of COVID infections in China (Jin et al., 2020) and in Europe (Gebhard et al., 2020). However, given the preference for boys over girls in many states of India, more developed states with lower poverty (e.g., Kerala) tend to have a higher sex ratio, and these states may have a better health system. Therefore, the effect of the sex ratio on the COVID‐19 may be ambiguous in India.^16^ We also control for the effect of the retail price of wheat to examine whether the food price has any association with the COVID‐19 pandemic. An increase in the wheat price may lead to the difficulty in accessing food or a macronutrient, but in the meantime, it may induce substitution into inferior cereals, such as *ragi* or maize, which may result in better nourishment (Gaiha et al., 2014). Our results are consistent with the latter hypothesis.

It is widely debated whether weather influences the COVID‐19 infection cases and/or linked to deaths. A study used the data on daily death numbers from Wuhan, China, in January–February 2020 and found that death counts are positively associated with temperature and negatively with relative humidity (Ma et al., 2020). We have collected the daily data on temperature, rainfall, and relative humidity from MERRA (Modern‐Era Retrospective analysis for Research and Applications—Version 2 web service) and have taken either week or month averages. It delivers time series of temperature (at 2m), relative humidity (at 2m), and rainfall. The data source is a NASA atmospheric reanalysis of the satellite era using the Goddard Earth Observing System Model (GEOS‐5) and focuses on historical climate analyses for a broad range of weather and climate time scales (GMAO, 2015). Due to the high correlation between rainfall and relative humidity, we use the variables $Temperature_{\mathrm{it}}$ and $Rainfall_{\mathrm{it}}$.

To capture the time and policy effects, we have included eight dummy variables for Lockdown Phases 2–4 and Unlock 1.0–5.0 for the weekly panel and seven monthly dummies for the monthly panel. This is aimed to capture the associations with the lockdown and unlock policies announced by the Government of India. Equation 1 has been estimated by pooled OLS with state and phase/month fixed effects, random‐effects model or mixed‐effects model (Bell & Jones, 2015), and random‐effects Tobit model with phase/month fixed effects.

## 5 RESULTS

We show the results of our regression analyses in Tables 2 and 3 corresponding to Equations 1 and 2. The main findings are summarized below.

**TABLE 2:** Correlates of cumulative severity ratio of COVID‐19

	(1)	(2)	(3)	(4)	(5)	(6)	(7)	(8)
Data‐ dependent variable	Weekly	Weekly	Monthly	Monthly	Monthly	Monthly	Monthly	Monthly
Level/first difference	FD cumulative	FD cumulative	Level cumulative	Level cumulative	Level cumulative	Level cumulative	FD cumulative	FD cumulative
	Severity ratio	Severity ratio	Severity ratio	Severity ratio	Severity ratio	Severity ratio	Severity ratio	Severity ratio
Model	Random effects^a^	Random effects	Random effects	Random effects	Tobit	Tobit	Random effects	Random effects
Explanatory variables	Est. coef.	Est. coef.	Est. coef.	Est. coef.	Est. coef.	Est. coef.	Est. coef.	Est. coef.
	(*Z* value)	(*Z* value)	(*Z* value)	(*Z* value)	(*Z* value)	(*Z* value)	(*Z* value)	(*Z* value)
Log per capita income	**0.329** ^b^, ^c^, ^e^		**4.473**		**5.599** ^c^		**1.955**	
	(5.04)***		(4.62)***		(3.83)***		(8.71)***	
Multi‐morbidity*	**0.025**		**0.536**		**0.669**		**0.215**	
(%)	(4.74)***		(3.13)***		(3.59)***		(6.50)***	
Rate of urbanization		0.01		**0.355**		**14.63**		**0.129**
(%)		(1.08)		(2.02)**		(1.83)*		(3.28)***
Sex ratio		**−0.00**		**−0.05**		**−0.06**		**−0.021**
		(4.01)***	(0.00)	(4.67)***	(0.00)	(2.75)***		(6.28)***
Log wheat prices (−1)	**−0.185**	**−0.18**	−1.781	−1.62	**−4.603**	**−4.47**	**−1.817**	**−1.758**
	(1.95)*	(1.88)*	(0.75)	(0.66)	(2.20)**	(2.08)**	(3.99)***	(3.81)***
Temperature	0.005	0.01	0.097	0.097	**0.173**	**0.178**	0.026	0.026
	(0.89)	(0.70)	(1.34)	(1.31)	(2.29)**	(2.25)**	(0.83)	(0.77)
Rainfall	−0.001	−0	−0.017	−0.01	−0.005	−0	**−0.026**	**−0.025**
[selective state dummies]^a^	(0.27)	(0.25)	(0.46)	(0.37)	(0.16)	(0.05)	(1.80)*	(1.69)*
D_Maharashtra^d^	**0.224**	**0.15**	**4.376**	−0.88	**5.521**	−0.25	**1.703**	−0.166
	(5.58)***	(1.77)*	(3.39)***	(0.59)	(3.64)***	(0.10)	(6.47)***	(0.41)
D_Andhra Pradesh	0.02	**0.25**	0.201	**2.2**	1.168	**3.776**	**0.47**	**1.463**
	(0.58)	(3.01)***	(0.20)	(1.85)*	(0.99)	(1.86)*	(2.47)**	(5.39)***
D_Assam	**0.19**	0.16	**3.368**	**6.476**	**4.875**	**12.85**	**1.601**	**2.605**
	(3.33)***	(1.44)	(2.50)**	(1.89)*	(2.82)***	(1.98)**	(6.12)***	(3.72)***
D_Gujarat	**−0.18**	**−0.22**	**−1.187**	**−4.84**	−1.328	**−5.44**	**−0.8**	**−2.086**
	(4.93)***	(3.91)***	(2.66)**	(2.89)***	(1.30)	(2.42)**	(4.15)***	(5.85)***
D_Kerala	**−0.933**	**0.44**	**−18.5**	**0.572**	**−21.65**	3.593	**−7.009**	1.313
	(6.53)***	(1.70)*	(4.05)***	**(0.26)**	(4.17)***	(0.65)	(7.93)***	(1.41)
D_Madhya Pradesh	**0.115**	**−0.07**	**2.218**	0.442	**3.062**	1.016	**0.888**	0.035
	(2.90)***	(1.99)**	(2.64)**	(0.54)	(2.24)**	(0.71)	(5.84)***	(0.17)
D_Rajasthan	0.065	−0.09	**2.018**	0.752	**2.776**	1.672	**0.685**	0.038
	(1.52)	(1.47)	(1.76)*	(0.57)	(1.90)*	(0.76)	(2.89)***	(0.11)
D_Tamil Nadu	**−0.127**	0.16	**−2.068**	−2.67	**−1.558**	−0.97	**−0.534**	−0.338
	(7.93)***	(0.97)	(4.57)***	(1.39)	(1.76)*	(0.24)	(5.32)***	(0.52)
D_Uttar Pradesh	**0.357**	−0.06	**5.826**	**6.314**	**6.341**	6.616	**2.011**	1.596
	(6.86)***	(0.27)	(7.69)***	(1.80)*	(3.47)***	(1.07)	(12.93)***	(1.62)
D_Lockdown Phase 2	0.013	0.02	**−4.049**	**−4.11**	**−4.947**	**−5.02**	**−0.914**	**−0.921**
(D_April)^g^	(0.57)	(0.67)	(4.71)***	(4.68)***	(9.22)***	(9.09)***	(2.84)***	(2.78)***
D_Lockdown Phase 3	0.045	0.05	**−4.06**	**−4.13**	**−4.791**	**−4.89**	**−0.833**	**−0.843**
(D_May)^g^	(1.03)	(1.11)	(4.20)***	(4.24)***	(7.80)***	(7.77)***	(2.41)**	(2.36)**
D_Lockdown Phase 4	−0.029	−0.03						
	(0.69)	(0.58)						
D_Unlock 1.0	0.044	0.05	**−3.781**	**−3.88**	**−4.335**	−4.47	−0.653	−0.683
(D_June)^g^	(0.91)	(0.87)	(4.03)***	(4.05)***	(7.18)***	(7.16)***	(1.87)*	(1.89)*
D_Unlock 2.0	0.082	0.08	**−3.386**	**−3.5**	**−3.906**	**−4.05**	−0.406	−0.434
(D_July)^g^	(1.48)	(1.42)	(4.02)***	(4.05)***	(6.72)***	(6.73)***	(1.17)	(1.21)
D_Unlock 3.0	**0.37**	**0.38**	**−2.278**	**−2.37**	**−2.618**	**−2.73**	0.354	0.346
(D_August)^g^	(1.98)**	(1.97)**	(3.63)***	(3.68)***	(4.50)***	(4.55)***	(0.58)	(0.55)
D_Unlock 4.0	**0.205**	**0.21**	**−0.961**	**−1.01**	**−1.187**	−1.25	0.52	0.52
(D_September)^g^	(3.65)***	(3.61)***	(2.31)**	(2.39)**	(2.31)**	(2.36)**	(0.97)	(0.94)
D_Unlock 5.0	**0.118**	**0.12**						
(October)^g^	(2.61)**	(2.62)**						
Constant	**−5.021**	**2.62**	**25.289**	**110.9**	**−2.267**	**69.03**	**−25.23**	**13.8**
	**(2.21)**	**(2.48)**	**(1.15)**	**(4.80)**	**(0.08)**	(1.83)*	(2.38)**	(1.92)
State fixed effects^a^	Yes	Yes	Yes	Yes	Yes	Yes	Yes	Yes
No of observations (*N*)	1,041	1,008	223	223	216	216	223	223
(left censored)					36	36		
No of states (*n*)	32	31	32	32	32	32	32	32
No of weeks (*T*)	32.5	32.5	7	7	7	7	7	7
Wald χ^2^	63.03	61.1	281	273.6	298.4	290.7	279.2	276
(*p*‐value)	(0.02)**	(0.02)**	(0.00)***	(0.00)***	(0.00)***	(0.00)***	(0.00)***	(0.00)***
*R* ^2^ within	0.0298	0.03	0.4416	0.446	‐	‐	0.184	0.186
*R* ^2^ between	1	1	1	1	‐	‐	1	1
*R* ^2^ overall	0.0571	0.06	0.6025	0.605	**‐**	**‐**	0.314	0.313
Breush and Pagan test	0	0	0	0	**‐**	**‐**	0	0
(*p*‐value)	(1.00)	(1.00)	(1.00)	(1.00)	**‐**	**‐**	(1.00)	(1.00)
Hausman test^f^	0	0	0	0	**‐**	**‐**	0	0
(*p*‐value)	(1.00)	(1.00)	(1.00)	(1.00)	‐	‐	(1.00)	(1.00)

^a^ State dummies or fixed effects for the other states have been included in all the cases. That is, the model has been estimated by a mixed effects model

^b^ *** = Significant at 1% level. ** = Significant at 5% level. * = significant at 105 level

^c^ The numbers in brackets show *Z* values, which are based on robust standard errors

^d^ D_ stands for a dummy variable (taking 1 or 0)

^e^ Statistically significant cases are highlighted as bold numbers

^f^ Hausman tests were carried out between FE and RE models.

^g^ Monthly dummies have been used instead of phase dummies in the case of monthly panel data.

**TABLE 3:** Correlates of COVID‐19 infection cases

	(9)	(10)	(11)	(12)	(13)	(14)	(15)	(16)
Data‐dependent variable	Weekly	Weekly	Weekly	Weekly	Monthly	Monthly	Monthly	Monthly
Level/first difference	Level	Level	Level	Level	Level	Level	Level	Level
	Cases	Cases	Cases	Cases	Cases	Cases	Cases	Cases
	(log)	(log)	(log)	(log)	(log)	(log)	(log)	(log)
Model	Random effects^a^	Random effects	Tobit	Tobit	Random effects^a^	Random effects	Tobit	Tobit
Explanatory variables	Est. Coef.	Est. Coef.	Est. Coef.	Est. Coef.	Est. Coef.	Est. Coef.	Est. Coef.	Est. Coef.
	(Z value)	(*Z* value)	(*Z* value)	(*Z* value)	(*Z* value)	(*Z* value)	(*Z* value)	(*Z* value)
Log per capita income	**−3.039** ^b^, ^c^, ^e^		**−3.016**		**−2.818** ^b^, ^c^		**−2.815**	
	(7.77)***		(8.33)***		(8.15)***		(4.73)***	
Multi‐morbidity*	**−0.278**		**−0.278**		**−0.28**		**−0.281**	
(%)	(4.48)***		(7.09)***		(3.20)***		(3.88)***	
Rate of urbanization		−0.127		**−0.13**		−0.154		−0.156
		(1.40)		(2.19)**		(1.21)		(1.58)
Sex ratio		**0.035**		**0.035**		**0.031**		**0.031**
		(5.22)***		(5.97)***		(4.25)***		(3.41)***
Log wheat prices (−1)	**−1.325**	**−1.301**	**−1.344**	**−1.32**	**−1.716**	**−1.678**	**−1.707**	**−1.668**
	(1.91)*	(1.86)*	(4.44)***	(4.29)***	(2.06)**	(1.98)**	(2.33)**	(2.22)**
Temperature	**0.135**	**0.133**	**0.138**	**0.136**	**0.168**	**0.17**	**0.169**	**0.171**
	(1.97)**	(1.87)*	(8.21)***	(7.75)***	(1.76)*	(1.71)*	(5.35)***	(5.18)***
Rainfall	**0.028**	**0.029**	**0.029**	**0.03**	**0.06**	**0.061**	**0.06**	**0.061**
[selective state dummies]^a^	(2.83)***	(2.84)***	(5.63)***	(5.65)***	(2.93)***	(2.97)***	(4.41)***	(4.40)***
D_Maharashtra^d^	**3.473**	**5.104**	**3.463**	**5.142**	**3.513**	**5.602**	**3.501**	**5.624**
	(6.50)***	(5.22)***	(10.00)***	(6.61)***	(4.58)***	(4.16)***	(5.72)***	(4.54)***
D_Andhra Pradesh	**1.604**	**−0.343**	**1.602**	**−0.314**	**1.674**	**0.022**	**1.668**	**0.026**
	(4.28)***	(0.72)	(5.81)***	(0.59)	(3.24)***	(0.05)	(3.49)***	(0.03)
D_Assam	**−2.839**	**−3.412**	**−2.831**	**−3.452**	**−2.43**	**−3.487**	**−2.434**	**−3.521**
	(7.00)***	(2.26)**	(7.02)***	(3.45)***	(5.31)***	**(1.62)**	(3.52)***	(2.04)**
D_Gujarat	**1.754**	**2.865**	**1.737**	**2.884**	**1.699**	**3.151**	**1.692**	**3.169**
	(4.17)***	(4.00)***	(6.09)***	(4.71)***	(2.94)***	(3.12)***	(3.77)***	(3.10)***
D_Kerala	**10.323**	**−2.629**	**10.33**	−2.516	**10.636**	−1.251	**10.67**	−1.206
	(6.20)***	**(1.18)**	(9.08)***	(1.48)	(4.52)***	(0.44)	(5.22)***	(0.46)
D_Madhya Pradesh	**−1.333**	0.216	**−1.324**	0.202	**−1.165**	0.149	**−1.167**	0.14
	(5.56)***	(0.47)	(3.72)***	(0.58)	(4.13)***	(0.23)	(2.03)**	(0.25)
D_Rajasthan	**−1.226**	0.059	**−1.226**	0.035	−1.117	−0.087	**−1.122**	−0.102
	(2.92)***	(0.07)	(3.40)***	(0.07)	(1.78)*	(0.08)	(1.85)*	(0.12)
D_Tamil Nadu	**3.998**	2.313	**3.994**	2.388	4.172	3.261	**4.169**	**3.297**
	(18.82)***	(1.45)	(16.48)***	(1.89)*	(13.66)***	(1.54)	(10.88)***	(1.67)*
D_Uttar Pradesh	**−5.905**	−3.391	**−5.888**	**−3.484**	**−5.876**	−4.453	**−5.874**	**−4.504**
	(22.66)***	(1.42)	(11.65)***	(2.10)**	(25.98)***	(1.35)	(7.45)***	(1.69)*
D_Lockdown Phase 2	**2.428**	2.448	**2.449**	**2.471**	**−7.748**	−7.768	−**7.754**	−**7.775**
(D_April)^g^	(7.27)***	(7.08)***	(13.18)***	(12.91)***	(15.07)***	(15.02)***	(34.98)***	(34.23)***
D_Lockdown Phase 3	**2.92**	**2.95**	**2.937**	**2.969**	**−6.62**	**−6.636**	**−6.625**	−6.641***
(D_May)^g^	(6.38)***	(6.21)***	(14.17)***	(13.90)***	(9.23)***	(9.14)***	(25.97)***	**(25.51)**
D_Lockdown Phase 4	**3.571**	**3.621**	**3.589**	**3.64**				
	(8.33)***	(8.15)***	(16.70)***	(16.44)***				
D_Unlock 1.0	**5.229**	**5.272**	**5.247**	**5.291**	**‐4.769**	**−4.78**	**−4.776**	**−4.784**
(D_June)^g^	(14.65)***	(14.12)***	(30.62)***	(29.78)***	(9.49)***	(9.29)***	(18.88)***	(18.38)***
D_Unlock 2.0	**6.282**	**6.291**	**6.3**	**6.31**	**−3.70**	**−3.747**	**−3.708**	**−3.755**
(D_July)^g^	(17.55)***	(16.86)***	(37.68)***	(36.43)***	(8.08)***	(7.96)***	(14.95)***	(14.68)***
D_Unlock 3.0	**7.493**	**7.51**	**7.511**	**7.528**	**−2.502**	**−2.541**	**−2.51**	−2.549**
(D_August)^g^	(19.66)***	(19.04)***	(44.89)***	(43.50)***	(6.26)***	(6.22)***	(10.15)***	**(10.02)**
D_Unlock 4.0	**8.666**	**8.699**	**8.689**	**8.723**	**−1.241**	**−1.251**	**−1.246**	**−1.256**
(D_September)^g^	(23.09)***	(22.64)***	(57.99)***	(56.50)***	(4.28)***	(4.23)***	(5.65)***	(5.56)***
D_Unlock 5.0	**9.67**	**9.708**	**9.703**	**9.741**				
(October)^g^	(22.53)***	(22.37)***	(66.05)***	(64.51)***				
Constant	3.108	−62.21	2.018	**−62.61**	1.557	−57.91	1.177	−58.13
	(0.13)	(4.52)	(0.29)	(8.36)	(0.05)	(2.76)	(0.10)	(4.55)
State fixed effects^a^	Yes	Yes	Yes	Yes	Yes	Yes	Yes	Yes
No of observations (*N*)	1,041	1,008	1,041	1,008	223	216	223	216
(left censored)			18	18			18	18
No of states (*n*)	32	31	32	31	32	31	32	31
No of weeks (*T*)	32.5	32.5	32.5	33.5	7	7	1	1
Wald χ^2^	11,714***	11,100***	11,700***	11,357***	4,740***	4,769***	4,149***	3,969***
*R* ^2^ within	0.8886	0.8875	‐	‐	0.9189	0.4538	‐	‐
*R* ^2^ between	1	1	‐	‐	1	1	‐	‐
*R* ^2^ overall	0.9219	0.9212	**‐**	**‐**	0.9494	0.6283	**‐**	**‐**
Breush and Pagan test	0	0			0	0		
(*p*‐value)	(1.00).	(1.00).			(1.00).	(1.00).		
Hausman test^f^	0	0			0	0		
(*p*‐value)	(1.00).	(1.00).			(1.00).	(1.00).		

^a^ State dummies or fixed effects for the other states have been included in all the cases. That is, the model has been estimated by a mixed effects model

^b^ *** = Significant at 1% level. ** = Significant at 5% level. * = significant at 105 level

^c^ The numbers in brackets show *Z* values, which are based on robust standard errors

^d^ D_ stands for a dummy variable (taking 1 or 0)

^e^ Statistically significant cases are highlighted as bold numbers

^f^ Hausman tests were carried out between FE and RE models

^g^ Monthly dummies have been used instead of phase dummies in the case of monthly panel data.

In Table 2, the results based on the weekly panel are shown in Columns 1 and 2, and those based on the monthly panels are in Columns 3–8. We find that log per capita income at the state level is positively associated with DCSR, weekly and monthly changes in CSR of COVID‐19 (a proxy for the development of the pandemic) (Columns 1, 3, 5, and 7) as well as monthly CSR, after controlling for state and phase/month fixed effects. For instance, an increase of 1% in per capita income is on average associated with an increase of 0.33% in the change of CSR (Column 1). As CSR is measured in percentages, not in the logarithm, this increase is substantial and implies that the state with a higher income tends to witness a faster change in CSR or a faster development of the pandemic. The reason is that a higher income level tends to be associated with more production, transportation, and movement of people and goods even in the lockdown phases. Consistent results are found for the monthly data. An increase of 1% in per capita income is associated with an increase of 4.4%–5.6% in CSR and a nearly 2% increase in the change in CSR on a monthly basis. If we replace CSR with DSR, we find that a 1% income increase is significantly associated with a 5.4% increase in DSR.^17^

We have also found some positive association between CSR or DCSR and multi‐morbidity for monthly data, but not weekly data. The estimated coefficient varies across different models, but, for instance, based on Column 4 on CSR, we observe that a 1% increase in urbanization is associated with a 0.36% increase in CSR as consistent with Das et al. (2021) and Olsen et al. (2020). As expected, we find that the share of those among the elderly with multi‐morbidity conditions is positively associated with DCSR or CSR (e.g., a 1% increase in the share is correlated with a 0.54%–0.67% increase in CSR, Columns 3 and 5). Similar results are found for DSR.

If the number of women per 1,000 men decreases by 1, this is on average associated with an increase of 0.05%–0.06% in CSR (or a 0.02 increase in DCSR). A consistent result has been found for DSR as well. However, the sign is reversed in Table 3. That is, a higher share of women is associated with a higher level of infection but lower level of fatalities. Whether this reflects any gender difference in the risks of infection and fatalities is not clear, but the results imply that demography is one of the important correlates of the COVID‐19 pandemic.

A lagged retail price of wheat is negatively correlated with DCSR or CSR, and the estimates are statistically significant in all the cases except Columns 3 or 4 (RE model applied to the monthly panel). Columns 1 and 2 based on the weekly data and 7 and 8 based on the monthly data show a similar level of parameter estimates. A 1% increase in wheat price is associated with a 0.17%–0.19% decrease in changes in CSR, while the estimated coefficient of the Tobit model suggests that a 1% price increase is correlated with a 4.5%–4.6% decrease in CSR (in levels). The results overall suggest a negative correlation between wheat prices and CSR, which could be due to shift to cheaper and more nutritious cereals. However, once we take the second or third lags, the parameter estimates are negative but not statistically significant.

We have controlled for temperature and rainfall to reflect the empirical literature on the correlates of the COVID‐19 infection and pandemic. While the estimated coefficient of temperature is positive and that of rainfall is negative in all the cases, we only find a positive and statistically significant estimate for temperature in Columns 5 and 6 (Tobit for CSR) and a negative and significant estimate for rainfall in Columns 7 and 8 (random‐effects model for DCSR). We refrain from inferring any associations between weather conditions and the pandemic once time and state effects are accounted for. Table 2 also shows coefficient estimates of state dummies for selected states. They do not necessarily match the rankings of CSR in Figure 1 or Figures A1 and A2 as estimated coefficients of state dummies have been derived after conditioning other covariates, such as per capita income. However, Maharashtra tends to have a higher parameter estimate when DCSR or CSR is statistically significant (e.g., Columns 1, 3, and 5). Phase or month dummies show that not only the level of CSR but also its change tends to increase in later periods, which implies that the pandemic has worsened over time. A decrease in DCSR from Unlock 4.0 to Unlock 5.0 (Columns 1 and 2) indicates that worsening of the pandemic slowed down in October 2020.

Table 3 shows the results on infection cases. Here log of infection cases, which has been found to be stationary based on the unit‐root tests, is used as a dependent variable in all the cases. Columns 9–12 based on the weekly panel, while Columns 13–16 on the monthly panel contain results when both random‐effects models and Tobit models are applied, given that there are some states with no cases at the onset of the pandemic. It is notable that many of the parameter estimates on CSR or DCSR in Table 2 are reversed in Table 3. For instance, log per capita income is negative and significant in all the cases.^18^ That is, if income increases by one percentage point, the number of cases tends to decrease by 2.8%–3.0% (with no causality implied by these results), after controlling for state fixed effects and phase/month dummies. Interpreting the results in Tables 2 and 3 together, a state with a higher income tends to experience the worse pandemic at relatively low case numbers on average. This is counter‐intuitive at first sight if we assume that income leads to more interactions among people leading to more cases, but we conjecture that a relatively rich state may be able to carry out more tests, but it does not necessarily have a capacity to cope with fatalities for a certain size of population.

On the contrary, the state with a higher share of the elderly with morbidity conditions tends to have lower COVID‐19 cases (where a 1% increase of the former is associated with a 0.28% decrease in the cases), while the unconditional correlation between the two variables is positive. It is conjectured that while morbidity conditions among the elderly can lead to fatalities once they are infected, they may not influence the probability of being infected at the population level. Urbanization is not significantly associated with the number of cases (except a negative and significant coefficient based on Tobit, Column 12). As noted earlier, the sex ratio is positive and significant, implying that the states with more females per 1,000 males tend to have more infection cases (an increase of one woman per 100 men is associated with a 0.03% increase in the cases). As in Table 2, retail prices of wheat are negatively correlated with the log of infection cases where a fall of 1% in wheat prices tends to lead to a decrease ranging from −1.3% to −1.7% in infection cases. It is conjectured that higher wheat price indices reflect a shift toward inferior but more nutritious cereals (Gaiha et al., 2014).^19^

On the effect of weather, both temperature and rainfalls have a positive and significant coefficient estimate, that is, hot and rainy weather conditions are correlated with higher COVID‐19 infection rates. A 1% increase in temperature is associated with a 0.13%–0.17% increase in the number of cases on average, other factors held constant. In contrast, a 1 mm increase in rainfall is associated with a 0.03%–0.06% increase in the cases. Phase or month dummy variables show that the number of cases tends to be larger and larger in subsequent months or phases. State dummy variables show, after controlling for covariates (e.g., income), that Maharashtra, Andhra Pradesh, Gujarat, Kerala, and Tamil Nadu are the states that exhibit a higher number of infection cases than other states.

## 6 CONCLUSIONS AND POLICY DISCUSSIONS

This study has provided econometric results on the socioeconomic, meteorological, and geographical correlates of the severity of COVID‐19 pandemic in India. We have used the measures of excess mortality called CSR and its first difference (DCSR) up to October 31, 2020. The log of COVID‐19 cases has also been estimated as a rapid increase in the cases implies a pandemic. The study has adopted a random‐effects model and a random‐effects Tobit model, the latter of which considers the fact that some states did not record COVID‐19‐oriented deaths in early phases. The factors associated with the severer pandemic reflected in a large CSR or DCSR include higher income at the state level, a higher share among the elderly with multi‐morbidity conditions, urbanization, a lower share of females in the population, lower local retail prices of wheat, and lockdown and unlock phases. On the contrary, the correlates of a higher number of infection cases—which are different from the above factors—include lower income, a lower share of the elderly with multi‐morbidity conditions, a higher share of females in the population, lower wheat prices, as well as hotter and/or rainier climatic conditions.

The positive association between the severity measures and per capita income implies that higher incomes are associated with higher mortalities. The underlying mechanisms include greater economic activity, more travel and intermixing, and, consequently, higher exposure to the infection and higher risk of dying if denied medical assistance. A negative association between the infection cases and per capita income is puzzling, but it could be the case that the state with greater economic activities did not necessarily have a matching capacity for testing.

A more or less expected result is the positive association between CSR (or DCSR) and urbanization. Although there has been a large‐scale reverse migration from urban areas to villages, indications are that large segments are forced to return to small towns and cities with flickering signs of economic revival.

The negative association of both CSR and DCSR with the sex ratio, the number of women per 1,000 men, means that the state with a higher share of women (i.e., a higher sex ratio) tends to have a lower severity ratio or its increase (or a milder pandemic) after controlling for state fixed effects. This is consistent with Joe et al. (2020) who argued that the evidence from various countries suggests that men are at greater risk of both infections and deaths, and that males are at a greater disadvantage than females with the CFR of 3.3% and 2.9%, respectively. In a statistical analysis, the authors show that the CFR among males is usually higher than females for most of the age groups.

A few limitations are briefly noted. First, as we have used a state, rather than a household, as the unit of analysis, we could not capture variation in COVID‐19 fatalities within states. Second, our analysis is limited by the lack of data on health capacity and infrastructure for measuring the response to the COVID pandemic. A third limitation is that we are unable to assess the impact of return migration on the villages/small towns to which they belong.

Finally, we will make a few points about COVID‐related policies^20^ and future research. First, we have shown a statistical association between the higher share among the elderly with multi‐morbidity conditions and the pandemic severity, while the development of COVID‐19 pandemic significantly differs across different states. It can be conjectured on the bases of our results and earlier studies on COVID‐19 (e.g., Joe et al., 2020) that the elderly suffering from NCDs are susceptible to the risk of dying from COVID‐19. Although our analysis has not been able to include the variables on health capacity and infrastructure, given under‐funding of the health sectors in a number of states, a case could be made to develop a fully integrated population‐based healthcare system that brings together the public and private sectors and the allopathic and indigenous systems, and is well coordinated at different levels of service delivery platforms—primary, secondary, and tertiary.^21^ It should address acute and chronic healthcare needs, offer accessible, good quality healthcare choices, and be cashless at the point of service delivery and would mitigate the severity of the pandemic. To understand the role of healthcare systems and funding in mitigating the severity of the pandemic, future research should examine the causal relationship between the health capacity and infrastructure and the COVID‐19 pandemic in India and elsewhere.

Another major concern is that the response to COVID‐19 infection depends on the immune system of the individuals. Although we did not include the variables on nutrition or health status at individual levels due to data constraints, we have shown that the development of the COVID‐19 pandemic is correlated with changes in wheat prices. The underlying mechanism connecting these two variables is unclear, but there exists a possibility that the change in wheat prices influences the nutritional conditions of the vulnerable groups in the population through changes in the availability of staple foods or substitution from wheat to other staple foods. Specifically, individuals with poor nutritional status are likely to have a weak immune system. A significant proportion of women in the age group 15–49 years, for example, are undernourished, and this makes them more vulnerable to COVID‐19 morbidity and mortality. As risks of chronic diseases accumulate over a life span, the old (60 years and above) tend to be more vulnerable to diabetes and cardiovascular diseases, and thus exposes them to higher risks of COVID‐19 morbidity and mortality. Amid elevated risks to lives and livelihoods, there is also a surge in hunger and food deprivation in both rural and urban areas. Besides, disruption of healthcare services is inimical to nutritional health (Joe et al., 2020). Therefore, food security is a major policy challenge (Reardon et al., 2020). Future research should focus on the causal link between food security or nutritional conditions of people and COVID‐19 morbidity or mortality.

As our results show that some states were slower to recover from the pandemic than others, a perceptive comment by Horton (2020) merits serious consideration, particularly when we look beyond the current pandemic. If we can diagnose new infections more rapidly, there is hope of exiting lockdown faster and more safely. For example, with self‐isolation when there are early signs of muscle pain, fatigue, headache, diarrhoea, and rashes, the likelihood of avoiding a second or third wave is greater. Another important observation is that prolonged lockdowns are not the answer to future waves of COVID‐19. Neither school closures are sustainable, nor could the economy be refrigerated again. What matters most is a mix of combination prevention that includes handwashing, respiratory hygiene, mask wearing, physical distancing, and avoiding mass gatherings, some of which were opted during the unlock phases. Future research should carry out rigorous evaluations of different social distancing policies and COVID‐19 morbidity or mortality using, for instance, phased‐implementations of lockdown or unlock policies.

In brief, the rapid surge of the corona pandemic calls for extraordinary measures. While some are identified here, their implementation is daunting.

## Data Availability

The authors have agreed to Wiley's Data Sharing Policies and are willing to share the data if the readers request. We are not willing to share the data in a public domain.

## ENDNOTES

^1^ Source: https://www.covid19india.org/ (accessed on 14 March 2021).

^2^ As of March 14, 2021 (https://www.covid19india.org/).

^3^ See Susskind and Vines (2020) for the comprehensive review.

^4^ The states are Andaman and Nicobar Islands, Andhra Pradesh, Arunachal Pradesh, Assam, Bihar, Chandigarh, Chhattisgarh, Delhi, Goa, Gujarat, Haryana, Himachal Pradesh, Jammu and Kashmir, Jharkhand, Karnataka, Kerala, Madhya Pradesh, Maharashtra, Manipur, Meghalaya, Mizoram, Nagaland, Odisha, Puducherry, Punjab, Rajasthan, Sikkim, Tamil Nadu, Telengana, Tripura, Uttar Pradesh, Uttarakhand, and West Bengal. The selection is determined by the availability of the data.

^5^ Based on Census 2011 estimates.

^6^ For instance, we interact dummy variables capturing different phases *of* lockdown policies with a state dummy (e.g., whether Maharashtra or not), but the causality rests on the assumption that the trend of the pandemic development is not different between Maharashtra and non‐Maharashtra. However, while this “parallel trend assumption” holds in a pre‐lockdown (March 15 to March 24) and Phase 1 (March 25 to April 14), it is unrealistic to assume that the macro‐environment is same for Maharashtra and other states, and there were no physical interactions to influence the pandemic development.

^7^ Outside India the rigorous studies of socioeconomic correlates of the COVID‐19 are scarce. Khalatbari‐Soltani et al., (2020) reviewed 29 studies across different countries, which reported the characteristics of patients with COVID‐19 and their potential risk factors, and only one study reported the occupational position of patients with mild or severe disease. The authors concluded that there is a need for the studies of socioeconomic factors associated with the COVID‐19 pandemic.

^8^ A question is whether the death numbers in 2017 would serve as a valid counterfactual. First, the national level death rate has been fairly stable and gradually declining from 7.4 to 7.3 deaths/1,000 population since 2012, and the year 2017 is not an exceptional year. Second, while India has experienced frequent and widespread droughts, there were no major droughts in 2017. The death numbers in 2017 would thus serve as a reasonable counterfactual for the present analysis of COVID‐19 (https://www.indexmundi.com/, accessed on 14 March 2021).

^9^ A full set of results will be provided on request.

^10^ However, the cases gradually declined in November and December in West Bengal in more recent periods not covered by this study.

^11^ We have also applied other alternatives of panel unit‐root tests and the results are broadly similar.

^12^ For the definitions of variables used, see Table A1.

^13^ The state‐wise estimates of daily confirmed COVID‐19 cases are taken from the official website of the Ministry of Health and Family Affairs, Government of India.

^14^ The data on state per capita incomes are obtained from the state economic surveys, and demographic data including population density and urban population are taken from the Census estimates.

^15^ The data on this variable are based on authors’ calculations from *The India Human Development Survey (IHDS*). IHDS is a nationally representative, multitopic panel survey of 41,554 households in 1,503 villages and 971 urban neighborhoods across India. The first round of interviews was completed in 2004–2005; and a second round of IHDS reinterviewed most of these households in 2011–2012. IHDS has been jointly organized by researchers from the University of Maryland and the National Council of Applied Economic Research (NCAER), New Delhi.

^16^ To avoid multicollinearity, one specification includes either “per capita income and multi‐morbidity” or “urbanization and the sex ratio” as covariates.

^17^ The results on DSR are not shown but will be provided on request.

^18^ The raw (unconditional) correlation coefficient between CSR and log of PCI is 0.153, while that between the log of cases and the log of PCI is −0.063.

^19^ We have carried out sensitivity tests for taking different lags of wheat prices. If we take the second lag of wheat price, the results are broadly similar. If we take the third lag, the sign is negative and statistically significant for the weekly panel and negative and nonsignificant for the monthly panel.

^20^ The points we make here (e.g., on health infrastructure) are not directly derived from our econometric results because of data constraints. However, these are, in our view, important in light of our study objectives and ongoing COVID‐19 pandemic where policymakers still face a great deal of uncertainty in policymaking (as of March 14, 2021).

^21^ Including the private sector, the total health expenditure as a percentage of GDP is estimated at 3.9%. Of the total expenditure, effectively about one‐third (30%) is contributed by the public sector. This contribution is low as compared to other developing and developed countries (Rao, 2018).

## 1

**TABLE A1:** Descriptive statistics

Variable	Weekly panel					Monthly panel				
	Obs.	Mean	Std. dev.	Min	Max	Obs.	Mean	Std. dev.	Min	Max
Cumulative severity ratio (CSR) of COVID−19 (%)	1,073	101.4	2.8	100.0	119.8	255	101.2	2.4	100.0	117.0
The first difference of CSR	1,041	0.1	0.7	−1.9	19.1	223	0.6	1.3	−6.0	9.4
log cumulative COVID infection cases	1,073	7.6	4.3	−6.9	14.3	255	7.0	4.5	−6.9	14.3
Log per capita income (Rs.)	1,073	11.5	0.5	10.3	12.9	255	11.5	0.5	10.3	12.9
Rate of multi‐morbidity	1,073	6.6	5.9	1.6	37.6	255	6.7	6.2	1.6	37.6
Rate of urbanization (%)	1,039	34.0	18.3	0.8	97.3	247	34.1	18.3	0.8	97.3
Sex ratio (no. of females per 1,000 males)	1,039	947.4	50.5	818.0	1,084.0	247	947.8	51.3	818.0	1,084.0
Log of retail price of wheat	1,073	3.3	0.2	3.0	4.1	255	3.4	0.2	3.0	4.1
Temperature	1,073	299.3	5.0	275.2	311.1	255	299.4	5.1	275.9	308.9
Rainfall	1,073	7.78	9.53	0	61.15	255	7.4	7.7	0.0	51.6
D_Lockdown Phase 2	1,073	0.08	0.24	0	1					
D_Lockdown Phase 3	1,073	0.06	0.20	0	1					
D_Lockdown Phase 4	1,073	0.06	0.20	0	1					
D_Unlock 1.0	1,073	0.11	0.30	0	1					
D_Unlock 2.0	1,073	0.12	0.32	0	1					
D_Unlock 3.0	1,073	0.12	0.32	0	1					
D_Unlock 4.0	1,073	0.18	0.38	0	1					
D_Unlock 5.0	1,073	0.15	0.36	0	1					
D_April						255	0.13	0.33	0	1
D_May						255	0.13	0.33	0	1
D_June						255	0.13	0.33	0	1
D_July						255	0.13	0.33	0	1
D_August						255	0.13	0.33	0	1
D_September						255	0.13	0.33	0	1
D_October						255	0.13	0.33	0	1
